# Inversion dimers dominate the crystal packing in the structure of trimethyl citrate (trimethyl 2-hy­droxy­propane-1,2,3-tri­carboxyl­ate)

**DOI:** 10.1107/S2056989018011222

**Published:** 2018-08-24

**Authors:** Rami Y. Morjan, Said M. El-Kurdi, Jannat N. Azarah, Neda A. Eleiwa, Omar S. Abu-Teim, Adel M. Awadallah, James Raftery, John M. Gardiner

**Affiliations:** a Department of Chemistry, Islamic University of Gaza, Gaza PO Box 108, Palestine; b Department of Chemistry, Al-Azhar University of Gaza, Gaza PO Box 1277, Palestine; c School of Chemistry, The University of Manchester, Brunswick Street, Manchester M13 9PL, UK; d Manchester Institute of Biotechnology, School of Chemistry and EPS, The University of Manchester, Manchester M1 7DN, UK

**Keywords:** crystal structure, trimethyl citrate, hydrogen bonds, inversion dimers, ring motifs

## Abstract

Trimethyl citrate, C_9_H_14_O_7_ (systematic name: trimethyl 2-hy­droxy­propane-1,2,3-tri­carboxyl­ate), **2**, was prepared by the esterification of citric acid and methanol in the presence of thionyl chloride at 273 K. The bond lengths and angles in **2** compare closely with those observed in citric acid. The C—C bonds adjacent to the terminal carboxyl groups are significantly shorter than those around the central C atom. The central carboxyl­ate group and the hy­droxy group occur in the normal planar arrangement with an r.m.s. deviation of 0.0171 Å from the mean plane involving all six atoms in the central unit. The crystal structure is almost completely dominated by the formation of inversion dimers through an O—H⋯O hydrogen bond, together with an extensive array of weaker C—H⋯O contacts. These generate a three-dimensional network structure with mol­ecules stacked along the *c*-axis direction.

## Abstract 2

The mol­ecular and crystal structure of trimethyl citrate is reported. The formation of inversion is the principal contributor to the crystal packing.

## Chemical context   

Esters of citric acid have received significant attention because of their many applications. Their use as plasticizers has grown because of their low toxicity, compatibility with the host materials and low volatility (Labrecque *et al.*, 1997 ▸; Garg *et al.*, 2014 ▸). They were investigated for use in degradable thermoset polymers (Halpern *et al.*, 2014 ▸). In the biological field, trimethyl citrate is used to synthesize citrate-functionalized ciprofloxacin conjugates and their anti­microbial activities have been determined against a panel of clinically-relevant bacteria (Md-Saleh *et al.*, 2009 ▸). Several different methods and catalysts have been employed for the synthesis of trimethyl citrate from citric acid and methanol using, for example, thionyl chloride (Ilewska & Chimiak, 1994 ▸) and zirconium(IV) dichloride oxide hydrate (Sun *et al.*, 2006 ▸). We report here the esterification of citric acid to form trimethyl citrate, **2**, together with its mol­ecular and crystal structure.

## Structural commentary   

**Figure 1:**
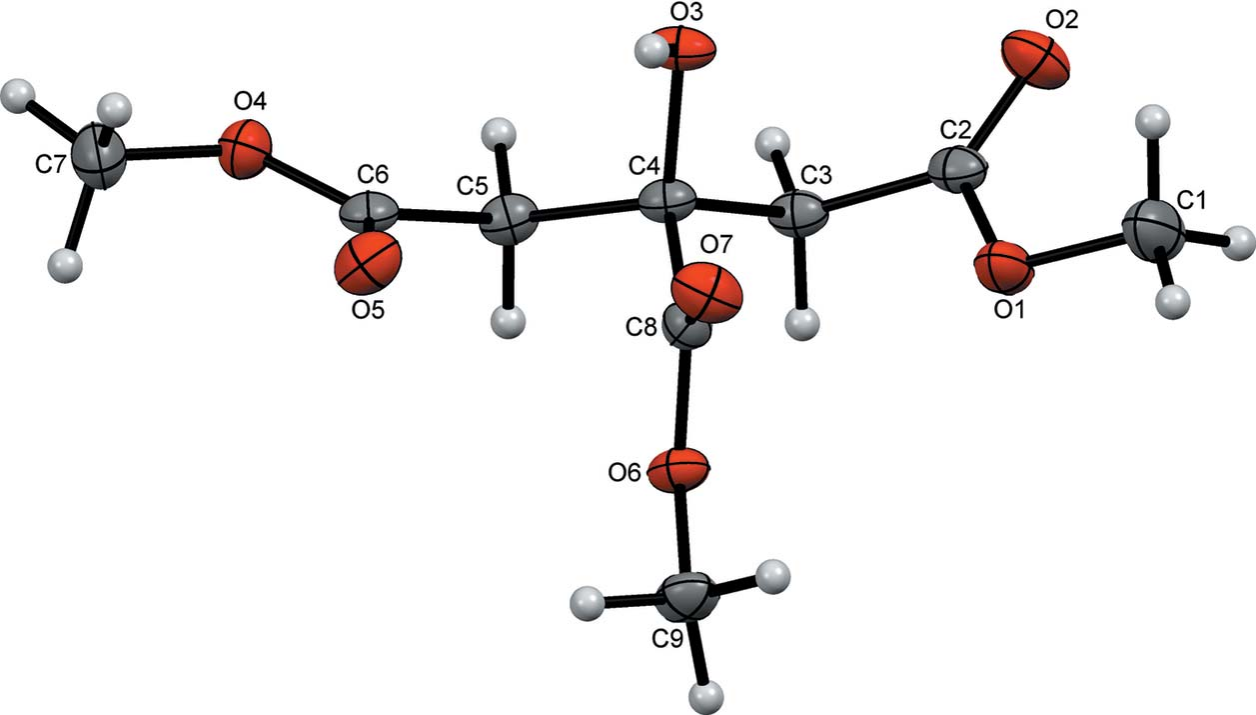
The structure of **2**, showing the atom numbering, with ellipsoids drawn at the 50% probability level.

The title compound, **2**, crystallizes in the triclinic space group *P* 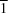, with one mol­ecule in the asymmetric unit. The mol­ecular structure of the compound, with the atom labelling, is shown in Fig. 1 ▸. The bond lengths and angles in **2** are comparable to those observed in citric acid, **1** (Glusker *et al.*, 1969 ▸; Roelofsen & Kanters, 1972 ▸; King *et al.*, 2011 ▸). The C2—C3 and C5—C6 bonds [1.506 (2) and 1.502 (2) Å, respectively] that bridge the outer terminal carboxyl groups are significantly shorter than those around the central C4 atom [C3—C4 = 1.5405 (19), C4—C5 = 1.5348 (19) and C4—C8 = 1.5398 (18) Å], an observation that mirrors what occurs in glycine itself. The carbonyl groups C2—O2, C8—O7 and C6—O5 are clearly double bonds with similar bond lengths [1.2046 (18), 1.2036 (18) and 1.2082 (18) Å, respectively]. Furthermore, the marked discrepancy between the C(=O)—O and O—Me distances, with the latter significantly longer in all instances, reflects considerable delocalization in the C(=O)—O units. This is again consistent with what is seen in other similar structures. The central carboxyl­ate group and the hydroxy group occur in the normal planar arrangement, with an O3—C4—C8—O6 torsion angle of −178.95 (11)° and an r.m.s. deviation of only 0.0171 Å from the best-fit mean plane through the O3, C4, C8, O7, O6 and C9 atoms.
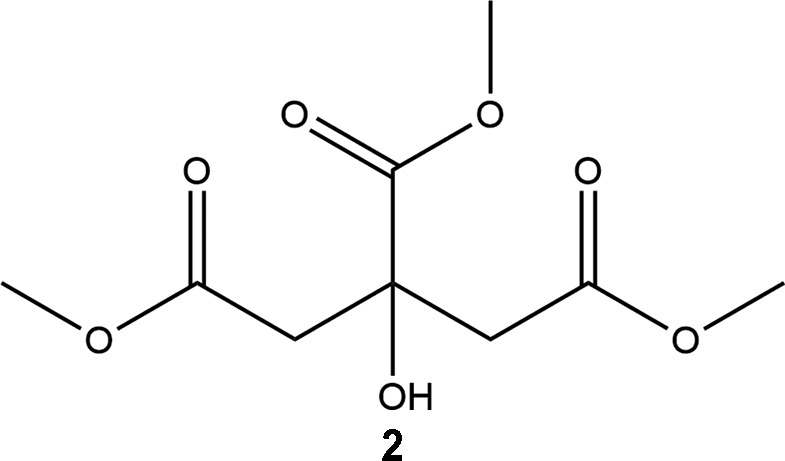


## Supra­molecular features   

**Figure 2:**
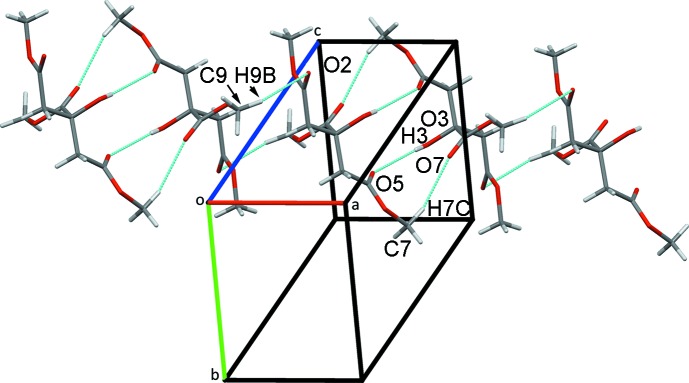
A view along *c* of chains of mol­ecules of **2** formed along (10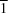) from pairs of inversion dimers.

**Figure 3:**
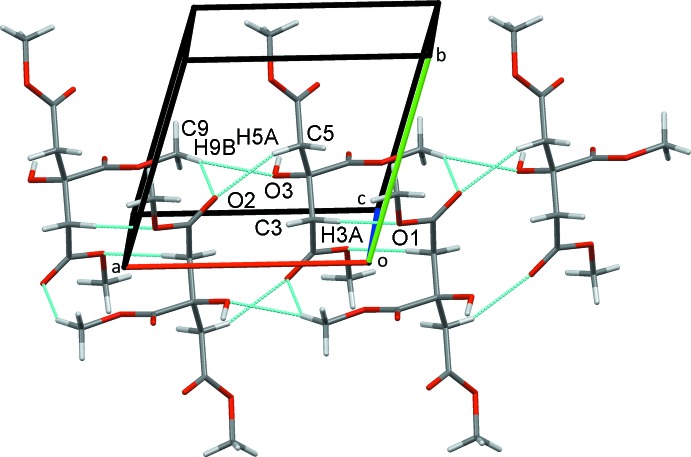
Chains of mol­ecules of **2** along the *a*-axis direction formed from pairs of inversion dimers.

**Figure 4:**
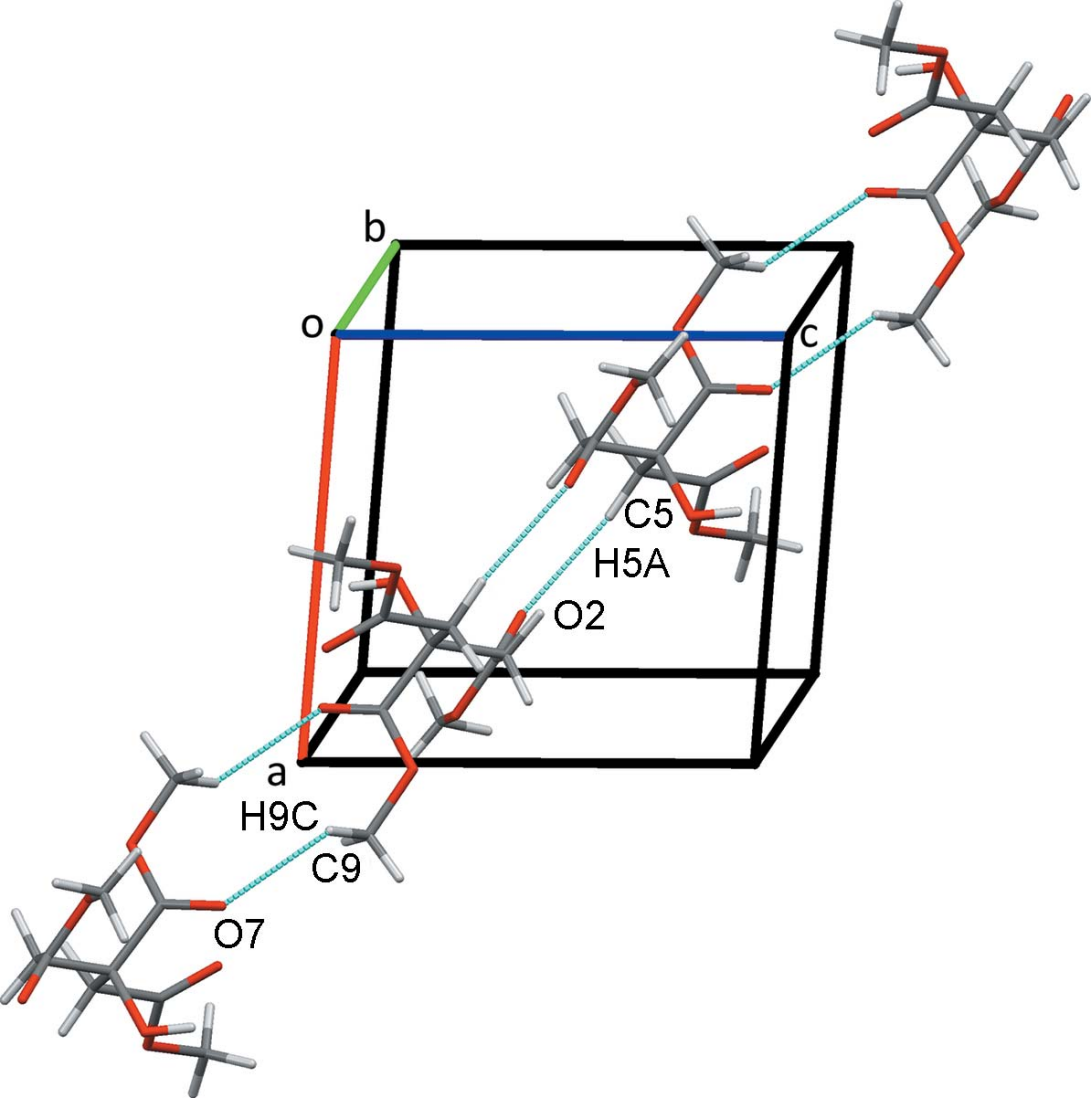
Pairs of inversion dimers that link mol­ecules of **2** into chains along the *ac* diagonal.

In the crystal, classical O3—H3⋯O5 hydrogen bonds form inversion dimers enclosing 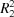
(12) rings. These contacts are supported by weaker inversion-related C7—H7*C*⋯O7^v^ hydrogen bonds with 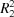
(16) ring motifs (Table 1 ▸). These dimers are linked into chains parallel to (10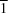) by inversion-related C9—H9*B*⋯O2^iii^ contacts that also form 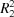
(16) rings (Fig. 2 ▸). H atoms from both of the methyl­ene groups in the mol­ecule are also involved in inversion-dimer formation. Pairs of C3—H3*A*⋯O1^iii^ hydrogen bonds enclose 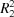
(8) rings that are linked by 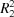
(12) ring C5—H5*A*⋯O2^iv^ inter­actions into chains along the *a*-axis direction. Weaker C9—H9*B*⋯O3^vi^ hydrogen bonds further stabilize these chains (Fig. 3 ▸). The 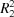
(12) ring C5—H5*A*⋯O2^iv^ inter­actions, mentioned previously, form more chains, this time linking another set of inversion dimers involving the 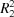
(10) ring C9—H9*C*⋯O7^vii^ contacts. These contacts form chains of dimers that run along the *ac* diagonal (Fig. 4 ▸).

**Figure 5:**
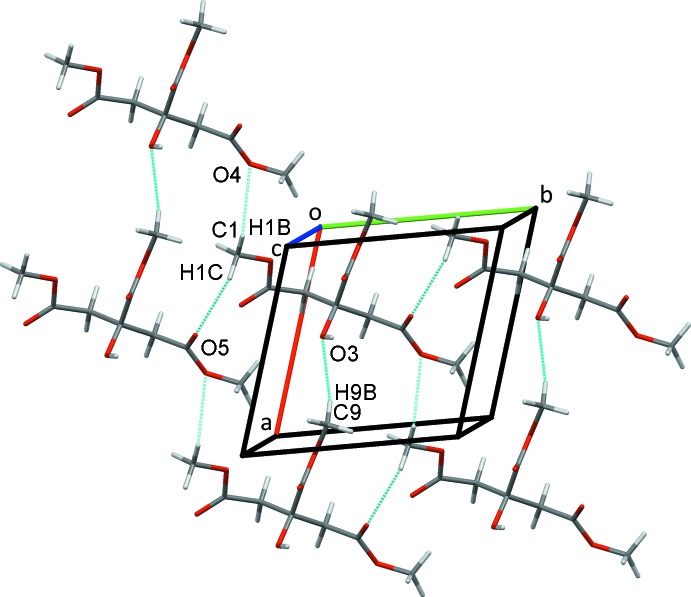
A view along *c* of the sheet of mol­ecules of **2** formed in the *ab* plane by weak C—H⋯O hydrogen bonds.

**Figure 6:**
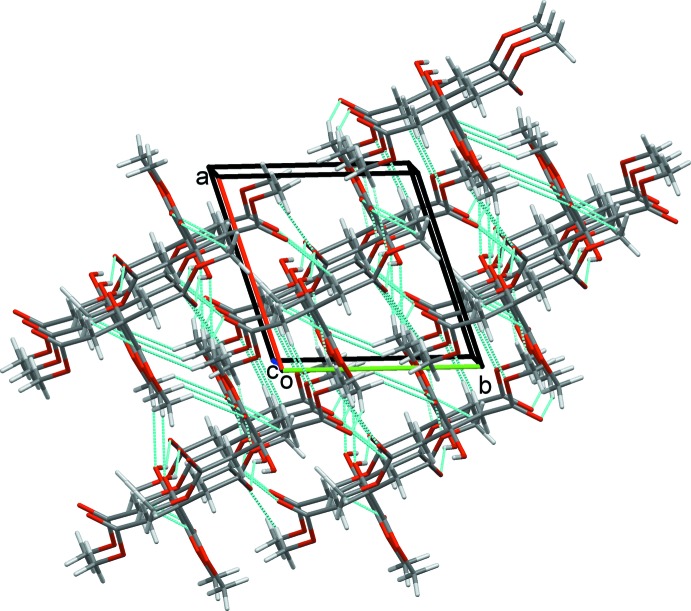
The overall packing of **2** viewed along the *c*-axis direction.

The only significant inter­molecular contacts in the crystal structure not to result in inversion-dimer formation involve weak C—H⋯O hydrogen bonds formed by the peripheral C1 and central C9 methyl groups. C1 acts as a bifurcated donor forming C1—H1*B*⋯O4^i^ and C1—H1*C*⋯O5^ii^ contacts that combine with C9—H9*B*⋯O3^vi^ hydrogen bonds to generate a sheet of mol­ecules in the *ab* plane (Fig. 5 ▸). Overall, this extensive array of both classical and nonclassical inter­molecular contacts generates a three-dimensional network structure with mol­ecules stacked along the *c*-axis direction (Fig. 6 ▸).

## Database survey   

A search of the Cambridge Structural Database (Version 5.39, updated February 2018; Groom *et al.*, 2016 ▸) for the title compound gave no hits. In contrast, a search for the O_2_CCH_2_C(O)(CO_2_)CH_2_CO_2_ fragment incorporating both organic and metal organic structures gave an impressive 404 hits. Limiting the search to organic structures, which eliminates the numerous metals salts of the citrate anions and the use of citrate as a ligand, reduced the hits to 124. In what follows, with few exceptions, only one or two recent examples of the plethora of different related systems are cited. The structure of citric acid itself has been reported several times, both in isolation (Glusker *et al.*, 1969 ▸) and as the monohydrate (Roelofsen & Kanters, 1972 ▸; King *et al.*, 2011 ▸). Eighteen examples of citric acid cocrystallized with various organic bases are also found (see, for example, Kerr *et al.*, 2016 ▸; Wang *et al.*, 2016 ▸). This search also revealed a lone neutral 1,5-di­methyl citrate (Li *et al.*, 2007*a* ▸) and a single monoanionic dimethyl citrate derivative, (−)-brucinium (*R*)-1,2-di­methyl­citrate hydrate (Bergeron *et al.*, 1997 ▸), with no related dianions. No examples of 1-methyl citrate or any of its anions were found, but 6-methyl citrate with the carboxyl­ate group on the central C atom has been reported (Li *et al.*, 2007*b* ▸; Aliyu *et al.*, 2009 ▸). In contrast, structures of more than 80 citrate anions have been reported; these included 48 monoanions with the proton lost from both the central (Inukai *et al.*, 2017 ▸; Wang *et al.*, 2017 ▸) and peripheral carboxyl­ate OH groups (Abraham *et al.*, 2016 ▸; Rammohan & Kaduk, 2016*a* ▸). Sixteen examples of citrate dianions (Rammohan & Kaduk, 2016*b* ▸, 2017*a* ▸) and 17 citrate trianions (Rammohan & Kaduk, 2017*b* ▸,*c* ▸) were also found.

## Synthesis and crystallization   

**Figure 7:**
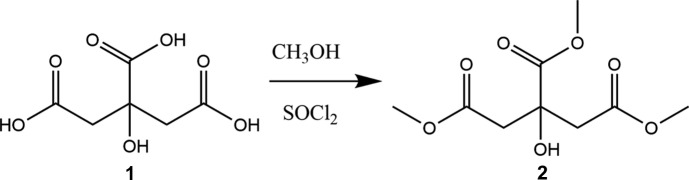
The synthesis of the title compound (**2**).

Citric acid (0.01 mol, 2.00 g) was dissolved in absolute methanol (50 mL) and the solution was cooled in an ice-bath under a nitro­gen atmosphere. To this solution, thionyl chloride (0.08 mol, 6.0 mL) was added dropwise with efficient stirring at 273 K for 1 h and the solution was left stirring overnight at 298 K (Fig. 7 ▸). The solvent was removed *in vacuo* and the solid residue was dissolved in ethyl acetate (15 mL), dried over MgSO_4_ and filtered. The solvent was removed under reduced pressure and the solid residue was purified by recrystallization from hexa­ne/ethyl acetate (1:3 *v*/*v*) to yield 1.6 g (80%) of the title compound as white crystals.

## Refinement   

Crystal data, data collection and structure refinement details are summarized in Table 2 ▸. Atom H3 of the OH group was located in a difference Fourier map and its coordinates refined with *U*_iso_(H) = 1.5*U*_eq_(O). The resulting O3—H3 distance of 0.80 (2) Å was acceptable. All H atoms bound to carbon were refined using a riding model, with C—H = 0.99 Å and *U*_iso_(H) = 1.2*U*_eq_(C) for CH_2_ H atoms, and C—H = 0.98 Å and *U*_iso_(H) = 1.5*U*_eq_(C) for CH_3_ H atoms.

## Supplementary Material

Crystal structure: contains datablock(s) I. DOI: 10.1107/S2056989018011222/sj5563sup1.cif

Structure factors: contains datablock(s) I. DOI: 10.1107/S2056989018011222/sj5563Isup2.hkl

Supporting information file. DOI: 10.1107/S2056989018011222/sj5563Isup3.cml

CCDC reference: 1500726

Additional supporting information:  crystallographic information; 3D view; checkCIF report

## Figures and Tables

**Table 1:** Hydrogen-bond geometry (Å, °)

*D*—H⋯*A*	*D*—H	H⋯*A*	*D*⋯*A*	*D*—H⋯*A*
C1—H1*B*⋯O4^i^	0.98	2.65	3.393 (2)	133
C1—H1*C*⋯O5^ii^	0.98	2.56	3.520 (2)	167
C3—H3*A*⋯O1^iii^	0.99	2.66	3.6388 (18)	170
C5—H5*A*⋯O2^iv^	0.99	2.51	3.4610 (18)	160
C7—H7*C*⋯O7^v^	0.98	2.53	3.3008 (19)	135
C9—H9*B*⋯O2^iii^	0.98	2.61	3.423 (2)	140
C9—H9*B*⋯O3^vi^	0.98	2.64	3.2576 (17)	121
C9—H9*C*⋯O7^vii^	0.98	2.61	3.4147 (19)	140
O3—H3⋯O5^v^	0.80 (2)	2.14 (2)	2.8428 (15)	147 (2)

Symmetry codes: (i) 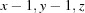; (ii) 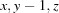; (iii) 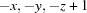; (iv) 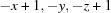; (v) 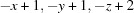; (vi) 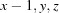; (vii) 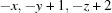.

**Table 2:** Experimental details

Crystal data	
Chemical formula	C_9_H_14_O_7_
*M*_r_	234.20
Crystal system, space group	Triclinic, *P* 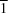
Temperature (K)	150
*a*, *b*, *c* (Å)	7.8428 (3), 8.0256 (3), 9.3965 (3)
α, β, γ (°)	109.915 (1), 92.832 (1), 104.493 (1)
*V* (Å^3^)	532.46 (3)
*Z*	2
Radiation type	Cu *K*α
μ (mm^−1^)	1.11
Crystal size (mm)	0.24 × 0.16 × 0.10

Data collection	
Diffractometer	Bruker APEXII CCD
Absorption correction	Multi-scan (*SADABS*; Bruker, 2001 ▸)
*T*_min_, *T*_max_	0.769, 0.897
No. of measured, independent and observed [*I* > 2σ(*I*)] reflections	4914, 1989, 1873
*R*_int_	0.030
(sin θ/λ)_max_ (Å^−1^)	0.618

Refinement	
*R*[*F* ^2^ > 2σ(*F* ^2^)], *wR*(*F* ^2^), *S*	0.038, 0.110, 1.08
No. of reflections	1989
No. of parameters	151
H-atom treatment	H atoms treated by a mixture of independent and constrained refinement
Δρ_max_, Δρ_min_ (e Å^−3^)	0.30, −0.28

Computer programs: *APEX2* (Bruker, 2003 ▸), *SAINT* (Bruker, 2003 ▸), *SHELXS97* (Sheldrick, 2008 ▸), *Mercury* (Macrae *et al.*, 2008 ▸), *SHELXL2018* (Sheldrick, 2015 ▸), *PLATON* (Spek, 2009 ▸), *publCIF* (Westrip 2010 ▸) and *WinGX* (Farrugia 2012 ▸).

## supplementary crystallographic information

### Crystal data

**Table:**

C_9_H_14_O_7_	*Z* = 2
*M**_r_* = 234.20	*F*(000) = 248
Triclinic, *P*1	*D*_x_ = 1.461 Mg m^−^^3^
*a* = 7.8428 (3) Å	Cu *K*α radiation, λ = 1.54178 Å
*b* = 8.0256 (3) Å	Cell parameters from 3559 reflections
*c* = 9.3965 (3) Å	θ = 5.1–72.3°
α = 109.915 (1)°	µ = 1.11 mm^−^^1^
β = 92.832 (1)°	*T* = 150 K
γ = 104.493 (1)°	Block, colourless
*V* = 532.46 (3) Å^3^	0.24 × 0.16 × 0.10 mm

### Data collection

**Table:**

Bruker APEXII CCD diffractometer	1873 reflections with *I* > 2σ(*I*)
Radiation source: X-ray, X-ray	*R*_int_ = 0.030
φ and ω scans	θ_max_ = 72.3°, θ_min_ = 5.1°
Absorption correction: multi-scan (SADABS; Bruker, 2001)	*h* = −9→9
*T*_min_ = 0.769, *T*_max_ = 0.897	*k* = −9→9
4914 measured reflections	*l* = −11→11
1989 independent reflections	

### Refinement

**Table:**

Refinement on *F*^2^	0 restraints
Least-squares matrix: full	Hydrogen site location: mixed
*R*[*F*^2^ > 2σ(*F*^2^)] = 0.038	H atoms treated by a mixture of independent and constrained refinement
*wR*(*F*^2^) = 0.110	*w* = 1/[σ^2^(*F*_o_^2^) + (0.0525*P*)^2^ + 0.2284*P*] where *P* = (*F*_o_^2^ + 2*F*_c_^2^)/3
*S* = 1.08	(Δ/σ)_max_ < 0.001
1989 reflections	Δρ_max_ = 0.30 e Å^−^^3^
151 parameters	Δρ_min_ = −0.28 e Å^−^^3^

### Special details

**Table:**

Geometry. All esds (except the esd in the dihedral angle between two l.s. planes) are estimated using the full covariance matrix. The cell esds are taken into account individually in the estimation of esds in distances, angles and torsion angles; correlations between esds in cell parameters are only used when they are defined by crystal symmetry. An approximate (isotropic) treatment of cell esds is used for estimating esds involving l.s. planes.

### Fractional atomic coordinates and isotropic or equivalent isotropic displacement parameters (Å^2^)

**Table:**

	*x*	*y*	*z*	*U*_iso_*/*U*_eq_	
C1	0.0409 (2)	−0.2623 (3)	0.7299 (2)	0.0381 (4)	
H1A	−0.005169	−0.376371	0.639926	0.057*	
H1B	−0.050722	−0.248183	0.796766	0.057*	
H1C	0.146568	−0.268417	0.785887	0.057*	
C2	0.22424 (19)	−0.0992 (2)	0.60115 (16)	0.0219 (3)	
C3	0.26796 (18)	0.0716 (2)	0.56095 (16)	0.0207 (3)	
H3A	0.160716	0.074354	0.502631	0.025*	
H3B	0.361749	0.065717	0.494144	0.025*	
C4	0.33242 (18)	0.25094 (19)	0.70328 (15)	0.0186 (3)	
C5	0.40405 (19)	0.4110 (2)	0.64816 (16)	0.0212 (3)	
H5A	0.503632	0.387793	0.590925	0.025*	
H5B	0.308690	0.413571	0.576515	0.025*	
C6	0.46821 (18)	0.5968 (2)	0.77536 (16)	0.0207 (3)	
C7	0.6601 (2)	0.8984 (2)	0.8491 (2)	0.0328 (4)	
H7A	0.565562	0.957176	0.844474	0.049*	
H7B	0.768047	0.965634	0.821830	0.049*	
H7C	0.685307	0.900853	0.953140	0.049*	
C8	0.18196 (18)	0.28329 (18)	0.79919 (16)	0.0196 (3)	
C9	−0.0983 (2)	0.3424 (2)	0.80083 (18)	0.0269 (3)	
H9A	−0.130073	0.261138	0.858908	0.040*	
H9B	−0.201720	0.322531	0.727756	0.040*	
H9C	−0.061107	0.471599	0.871360	0.040*	
O1	0.08823 (14)	−0.10558 (15)	0.68144 (14)	0.0288 (3)	
O2	0.30055 (15)	−0.21698 (16)	0.56613 (13)	0.0314 (3)	
O3	0.47114 (14)	0.22946 (16)	0.79070 (12)	0.0238 (3)	
H3	0.472 (3)	0.284 (3)	0.880 (3)	0.036*	
O4	0.60317 (14)	0.70848 (14)	0.74259 (12)	0.0262 (3)	
O5	0.40234 (14)	0.64142 (15)	0.89107 (12)	0.0286 (3)	
O6	0.04717 (13)	0.30097 (14)	0.71842 (11)	0.0220 (3)	
O7	0.18789 (15)	0.29190 (16)	0.92982 (12)	0.0277 (3)	

### Atomic displacement parameters (Å^2^)

**Table:**

	*U*^11^	*U*^22^	*U*^33^	*U*^12^	*U*^13^	*U*^23^
C1	0.0306 (9)	0.0363 (9)	0.0600 (12)	0.0126 (7)	0.0154 (8)	0.0297 (9)
C2	0.0185 (7)	0.0249 (7)	0.0202 (7)	0.0083 (6)	−0.0007 (6)	0.0046 (6)
C3	0.0189 (7)	0.0253 (7)	0.0174 (7)	0.0085 (6)	0.0025 (5)	0.0056 (6)
C4	0.0160 (6)	0.0241 (7)	0.0160 (6)	0.0075 (5)	0.0018 (5)	0.0064 (5)
C5	0.0198 (7)	0.0260 (7)	0.0179 (7)	0.0071 (6)	0.0045 (5)	0.0076 (6)
C6	0.0164 (6)	0.0254 (7)	0.0209 (7)	0.0074 (5)	−0.0007 (5)	0.0088 (6)
C7	0.0347 (9)	0.0226 (8)	0.0398 (9)	0.0053 (6)	0.0003 (7)	0.0123 (7)
C8	0.0194 (7)	0.0188 (6)	0.0200 (7)	0.0062 (5)	0.0039 (6)	0.0055 (5)
C9	0.0188 (7)	0.0300 (8)	0.0304 (8)	0.0102 (6)	0.0073 (6)	0.0065 (6)
O1	0.0232 (5)	0.0287 (6)	0.0427 (7)	0.0120 (4)	0.0118 (5)	0.0189 (5)
O2	0.0350 (6)	0.0325 (6)	0.0339 (6)	0.0205 (5)	0.0107 (5)	0.0124 (5)
O3	0.0214 (5)	0.0358 (6)	0.0168 (5)	0.0152 (4)	0.0022 (4)	0.0078 (4)
O4	0.0247 (5)	0.0243 (5)	0.0306 (6)	0.0053 (4)	0.0054 (4)	0.0121 (4)
O5	0.0255 (6)	0.0327 (6)	0.0213 (5)	0.0058 (5)	0.0045 (4)	0.0037 (4)
O6	0.0166 (5)	0.0272 (5)	0.0212 (5)	0.0093 (4)	0.0026 (4)	0.0054 (4)
O7	0.0326 (6)	0.0366 (6)	0.0221 (5)	0.0174 (5)	0.0112 (4)	0.0145 (5)

### Geometric parameters (Å, º)

**Table:**

C1—O1	1.4497 (19)	C5—H5B	0.9900
C1—H1A	0.9800	C6—O5	1.2083 (18)
C1—H1B	0.9800	C6—O4	1.3279 (18)
C1—H1C	0.9800	C7—O4	1.4494 (19)
C2—O2	1.2047 (18)	C7—H7A	0.9800
C2—O1	1.3372 (18)	C7—H7B	0.9800
C2—C3	1.506 (2)	C7—H7C	0.9800
C3—C4	1.5405 (19)	C8—O7	1.2036 (18)
C3—H3A	0.9900	C8—O6	1.3352 (17)
C3—H3B	0.9900	C9—O6	1.4537 (17)
C4—O3	1.4107 (16)	C9—H9A	0.9800
C4—C5	1.5348 (19)	C9—H9B	0.9800
C4—C8	1.5398 (18)	C9—H9C	0.9800
C5—C6	1.502 (2)	O3—H3	0.80 (2)
C5—H5A	0.9900		
			
O1—C1—H1A	109.5	C4—C5—H5B	108.8
O1—C1—H1B	109.5	H5A—C5—H5B	107.7
H1A—C1—H1B	109.5	O5—C6—O4	124.07 (14)
O1—C1—H1C	109.5	O5—C6—C5	124.44 (13)
H1A—C1—H1C	109.5	O4—C6—C5	111.46 (12)
H1B—C1—H1C	109.5	O4—C7—H7A	109.5
O2—C2—O1	123.32 (14)	O4—C7—H7B	109.5
O2—C2—C3	125.04 (13)	H7A—C7—H7B	109.5
O1—C2—C3	111.64 (12)	O4—C7—H7C	109.5
C2—C3—C4	112.59 (11)	H7A—C7—H7C	109.5
C2—C3—H3A	109.1	H7B—C7—H7C	109.5
C4—C3—H3A	109.1	O7—C8—O6	125.36 (13)
C2—C3—H3B	109.1	O7—C8—C4	123.63 (13)
C4—C3—H3B	109.1	O6—C8—C4	111.01 (11)
H3A—C3—H3B	107.8	O6—C9—H9A	109.5
O3—C4—C5	110.16 (11)	O6—C9—H9B	109.5
O3—C4—C8	109.64 (11)	H9A—C9—H9B	109.5
C5—C4—C8	111.26 (11)	O6—C9—H9C	109.5
O3—C4—C3	106.41 (11)	H9A—C9—H9C	109.5
C5—C4—C3	107.72 (11)	H9B—C9—H9C	109.5
C8—C4—C3	111.52 (11)	C2—O1—C1	115.46 (12)
C6—C5—C4	113.73 (11)	C4—O3—H3	109.9 (15)
C6—C5—H5A	108.8	C6—O4—C7	115.72 (12)
C4—C5—H5A	108.8	C8—O6—C9	115.65 (11)
C6—C5—H5B	108.8		
			
O2—C2—C3—C4	−116.42 (15)	C5—C4—C8—O7	−120.73 (15)
O1—C2—C3—C4	63.70 (15)	C3—C4—C8—O7	118.97 (15)
C2—C3—C4—O3	51.89 (14)	O3—C4—C8—O6	−178.93 (11)
C2—C3—C4—C5	170.01 (11)	C5—C4—C8—O6	58.96 (15)
C2—C3—C4—C8	−67.63 (15)	C3—C4—C8—O6	−61.34 (15)
O3—C4—C5—C6	−65.67 (14)	O2—C2—O1—C1	2.2 (2)
C8—C4—C5—C6	56.14 (15)	C3—C2—O1—C1	−177.89 (13)
C3—C4—C5—C6	178.65 (11)	O5—C6—O4—C7	−5.5 (2)
C4—C5—C6—O5	−34.35 (19)	C5—C6—O4—C7	172.48 (12)
C4—C5—C6—O4	147.68 (12)	O7—C8—O6—C9	3.0 (2)
O3—C4—C8—O7	1.38 (19)	C4—C8—O6—C9	−176.68 (11)

### Hydrogen-bond geometry (Å, º)

**Table:**

*D*—H···*A*	*D*—H	H···*A*	*D*···*A*	*D*—H···*A*
C1—H1*B*···O4^i^	0.98	2.65	3.393 (2)	133
C1—H1*C*···O5^ii^	0.98	2.56	3.520 (2)	167
C3—H3*A*···O1^iii^	0.99	2.66	3.6388 (18)	170
C5—H5*A*···O2^iv^	0.99	2.51	3.4610 (18)	160
C7—H7*C*···O7^v^	0.98	2.53	3.3008 (19)	135
C9—H9*B*···O2^iii^	0.98	2.61	3.423 (2)	140
C9—H9*B*···O3^vi^	0.98	2.64	3.2576 (17)	121
C9—H9*C*···O7^vii^	0.98	2.61	3.4147 (19)	140
O3—H3···O5^v^	0.80 (2)	2.14 (2)	2.8428 (15)	147 (2)

Symmetry codes: (i) *x*−1, *y*−1, *z*; (ii) *x*, *y*−1, *z*; (iii) −*x*, −*y*, −*z*+1; (iv) −*x*+1, −*y*, −*z*+1; (v) −*x*+1, −*y*+1, −*z*+2; (vi) *x*−1, *y*, *z*; (vii) −*x*, −*y*+1, −*z*+2.

## References

[bb1] Abraham, A., Apperley, D. C., Byard, S. J., Ilott, A. J., Robbins, A. J., Zorin, V., Harris, R. K. & Hodgkinson, P. (2016). *CrystEngComm*, **18**, 1054–1063.

[bb2] Aliyu, L., Mohamed, N., Quah, C. K. & Fun, H.-K. (2009). *Acta Cryst.* E**65**, o1843. 10.1107/S1600536809025598 PMC2977126 21583543

[bb3] Bergeron, J. R., Xin, M., Smith, E. R., Wollenweber, M., McManis, S. R., Ludin, C. & Abboud, A. K. (1997). *Tetrahedron*, **53**, 427–434.

[bb5] Bruker (2001). *SADABS*. Bruker AXS Inc., Madison, Wisconsin, USA.

[bb6] Bruker (2003). *APEX2*, *SMART* and *SAINT-Plus*. Bruker AXS Inc., Madison, Wisconsin, USA.

[bb7] Farrugia, L. J. (2012). *J. Appl. Cryst.* **45**, 849–854.

[bb8] Garg, B., Bisht, T. & Ling, Y. C. (2014). *RSC Adv.* **4**, 57297–57307.

[bb9] Glusker, J. P., Minkin, J. A. & Patterson, A. L. (1969). *Acta Cryst.* B**25**, 1066–1072. 10.1107/s0567740869003542 5819635

[bb10] Groom, C. R., Bruno, I. J., Lightfoot, M. P. & Ward, S. C. (2016). *Acta Cryst.* B**72**, 171–179. 10.1107/S2052520616003954 PMC4822653 27048719

[bb11] Halpern, J. M., Urbanski, R., Weinstock, A. K., Iwig, D. F., Mathers, R. T. & von Recum, H. A. (2014). *J. Biomed. Mater. Res. Part A*, **102**, 1467–1477. 10.1002/jbm.a.34821 PMC3972072 23737239

[bb12] Ilewska, M. J. & Chimiak, A. (1994). *Amino Acids*, **7**, 89–96. 10.1007/BF00808450 24185977

[bb13] Inukai, K., Takiyama, K., Noguchi, S., Iwao, Y. & Itai, S. (2017). *Int. J. Pharm.* **521**, 33–39. 10.1016/j.ijpharm.2017.01.065 28196716

[bb14] Kerr, H. E., Mason, H. E., Sparkes, H. A. & Hodgkinson, P. (2016). *CrystEngComm*, **18**, 6700–6707.

[bb15] King, M. D., Davis, E. A., Smith, T. M. & Korter, T. M. (2011). *J. Phys. Chem. A*, **115**, 11039–11044. 10.1021/jp204750v 21923096

[bb16] Labrecque, L. V., Kumar, R. A., Dave, V., Gross, R. A. & McCarthy, S. P. (1997). *J. Appl. Polym. Sci.* **66**, 1507–1513.

[bb17] Li, M., Wang, Y., Fu, D. & Liu, X. (2007*b*). *Acta Cryst.* E**63**, o4497.

[bb18] Li, M., Wang, Y., Ma, P., Fu, D. & Liu, X. (2007*a*). *Acta Cryst.* E**63**, o4632.

[bb19] Macrae, C. F., Bruno, I. J., Chisholm, J. A., Edgington, P. R., McCabe, P., Pidcock, E., Rodriguez-Monge, L., Taylor, R., van de Streek, J. & Wood, P. A. (2008). *J. Appl. Cryst.* **41**, 466–470.

[bb20] Md-Saleh, S. R., Chilvers, E. C., Kerr, K. G., Milner, S. J., Snelling, A. M., Weber, J. P., Thomas, G. H., Duhme-Klair, A. K. & Routledge, A. (2009). *Bioorg. Med. Chem. Lett.* **19**, 1496–1498. 10.1016/j.bmcl.2009.01.007 19179071

[bb21] Rammohan, A. & Kaduk, J. A. (2016*a*). *Acta Cryst.* E**72**, 854–857. 10.1107/S2056989016008343 PMC4908552 27308058

[bb22] Rammohan, A. & Kaduk, J. A. (2016*b*). *Acta Cryst.* E**72**, 170–173. 10.1107/S2056989016000232 PMC4770952 26958380

[bb23] Rammohan, A. & Kaduk, J. A. (2017*a*). *Acta Cryst.* E**73**, 92–95. 10.1107/S2056989016020168 PMC5209781 28083145

[bb24] Rammohan, A. & Kaduk, J. A. (2017*b*). *Acta Cryst.* E**73**, 250–253. 10.1107/S2056989017001086 PMC5290576 28217353

[bb25] Rammohan, A. & Kaduk, J. A. (2017*c*). *Acta Cryst.* E**73**, 286–290. 10.1107/S2056989017001256 PMC5290583 28217360

[bb26] Roelofsen, G. & Kanters, J. A. (1972). *Cryst. Struct. Commun.* **1**, 23–26.

[bb27] Sheldrick, G. M. (2008). *Acta Cryst.* A**64**, 112–122. 10.1107/S0108767307043930 18156677

[bb28] Sheldrick, G. M. (2015). *Acta Cryst.* C**71**, 3–8.

[bb29] Spek, A. L. (2009). *Acta Cryst.* D**65**, 148–155. 10.1107/S090744490804362X PMC2631630 19171970

[bb30] Sun, H. B., Hua, R. M. & Yin, Y. W. (2006). *Molecules*, **11**, 263–271. 10.3390/11040263 PMC6148516 17962757

[bb31] Wang, L., Guo, M., Jin, S., Sun, L., Wang, Y., Xu, W. & Wan, D. (2016). *J. Chem. Crystallogr.* **46**, 399–410.

[bb32] Wang, C., Paul, S., Wang, K., Hu, S. & Sun, C. C. (2017). *Cryst. Growth Des.* **17**, 6030–6040.

[bb33] Westrip, S. P. (2010). *J. Appl. Cryst.* **43**, 920–925.

